# Supplementary material for: CX3CL1 Action on Microglia Protects from Diet-Induced Obesity by Restoring POMC Neuronal Excitability and Melanocortin System Activity Impaired by High-Fat Diet Feeding
Source: Int J Mol Sci. 2022 Jun 7;23(12):6380. doi: 10.3390/ijms23126380 (PMC9224384; doi:10.3390/ijms23126380)
Supplement: Supplementary file 1 [file ijms-23-06380-s001.zip › ijms-1743561-supplementary.pdf]

Supplementary Figure S1:

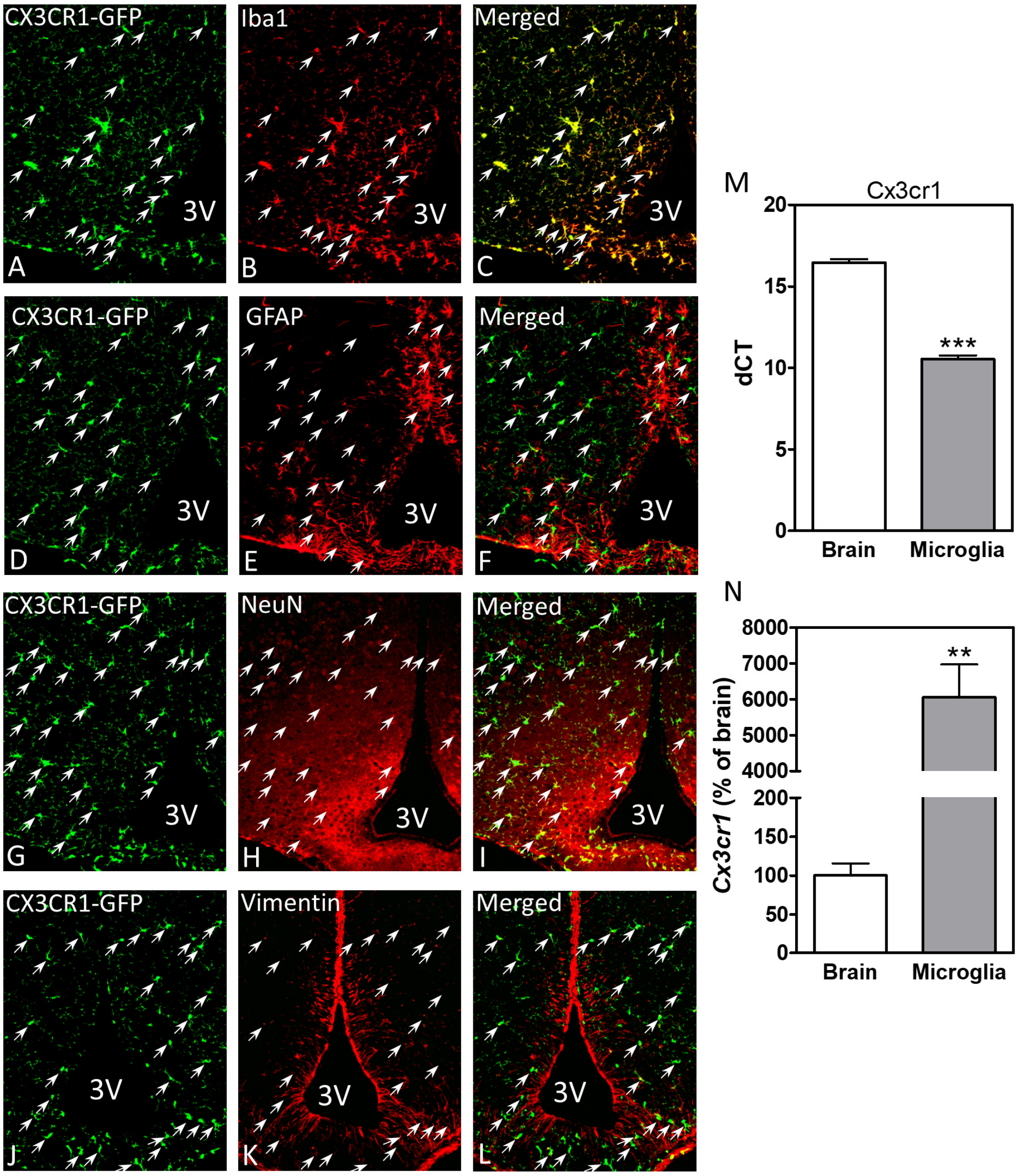

Supplementary Figure S1. CX3CR1 is expressed in microglia, but not astrocytes, neurons or tanycytes in the MBH. Representative images showing CX3CR1-GFP (A, D, G, J) and Iba1 (B), GFAP (E), NeuN (H), Vimentin (K) immunoreactivity in the MBH. (C) All CX3CR1-GFP positive cells are also positive for the microglial marker Iba1. There is no colocalization between CX3CR1-GFP and the astrocytic marker GFAP (F), the neuronal marker NeuN (I) or the tanycyte marker vimentin (L). Delta Ct values (M) and relative expression (N) of *Cx3cr1* expressed in whole brain and sorted brain microglia. ** $p < 0.01$; *** $p < 0.001$.
